# What travel scenarios are the opportunities of car sharing?

**DOI:** 10.1371/journal.pone.0260605

**Published:** 2021-12-09

**Authors:** Yan Xu, Xuehong Ji, Ziniu Jin

**Affiliations:** Economic and Management School, North China University of Technology, Beijing, China; Central South University, CHINA

## Abstract

In face of high-investment and low-revenue crisis, it is critical for new-born car-sharing companies to focus on niche market. Existing literatures have already discussed the niche market of car sharing based on people’s irregular travel demands and scenarios; however, current research findings are still lack of adequate travel data support. Aiming to solve this problem, we develop a travel scenarios mining method that first define land usage types of travel Origin-Destination (OD) locations using spatial clustering analysis of city’s Points of interests (POI) data, and then discover the most representative travel scenarios using association rules mining method with car-sharing records data. Applying this approach to the car-sharing service case in Beijing, China, we find that: *day-time business trips and evening entertainment trips around the city centre*, *commuting trips in off-peak time*, and *short-distance city travel for tourism* are three travel scenarios suitable for car sharing’s niche in the initial stage of market entering. Furthermore, spatio-temporal consumption profiles and competitive advantages of car sharing in the three travel scenarios are analyzed. Finally, theoretical and managerial implications are discussed. In this study, we address the question of finding niche market and suggest that it is critical for car sharing industry treading through the crisis in the early stage of development.

## Background

Car sharing (timeshare rental cars) system offers an alternative to car ownership [1]. Using the mobile application, customers are able to find a car, pay the rent fee, and verify if the car reaches the parking place. After the end of the lease, customers can park the car at any place of the service region in the city to enable another user nearby to use it.

Sharing can improve car usage and reduce parking cost [2, 3]. Moreover, most car-sharing systems provide services with electric car fleet [4–9]. Therefore, such system can alleviate urban problems such as excessive vehicle ownership [10–12], limited parking space [13], and environmental degradation [13, 14]. Attractive rental economics can facilitate electric vehicle penetration, thereby reducing petroleum consumption and emissions [5, 15–20]. Electric car-sharing system (ECSS) has gained popularity worldwide. For instance, Autolib operates a 2500 car fleet in Paris; Car2go owns a fleet of nearly 14,000 vehicles in North America, Europe, and Asia [21]; Evcard operates a more than 50000 car fleet in 65 cities of China. Car sharing offers an alternative to match highly dispersed travel demand and enhance travel experience in some scenarios [13, 22].

**Fig 1:**
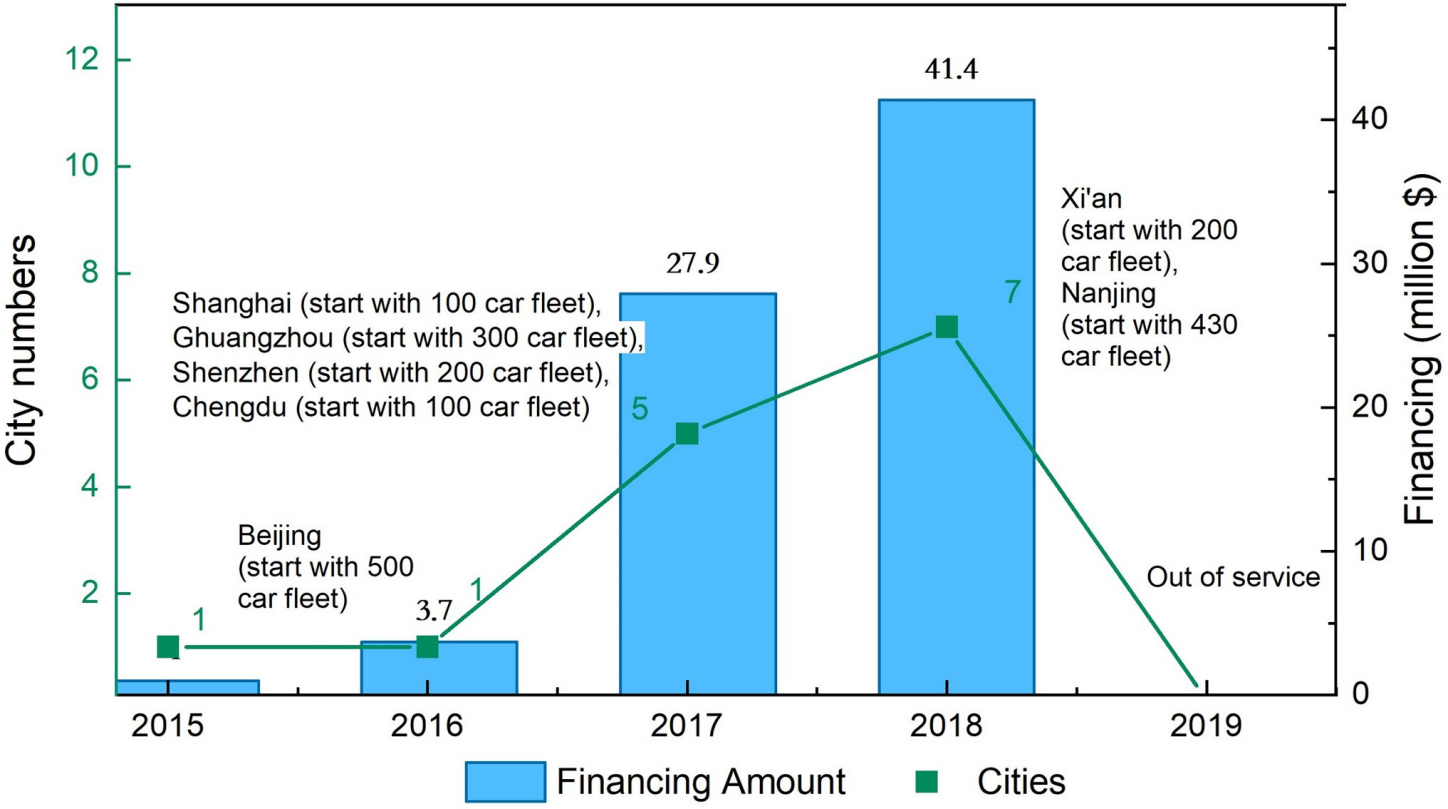
Financing and expansion process of Togo in China (data source: Network data collection).

Despite these advantages, the new business faces severe challenges [9, 14, 23]. Car-sharing platform with more rental sites and larger car fleet can offer more user flexibility; however, this would require greater investment. Car-sharing companies have been struggling to balance usage and profit rates. For instance, Car2go, the largest car-sharing platform in North America, announced that it would shut down service in San Diego in the end of 2016. Because it was unable to attract more city residents, leaving the programme unprofitable [24]. Autolib closed its business in July 2018. The company had the largest car-sharing scheme in Europe, owning 2,500 operational vehicles and over 150,000 subscribers. Furthermore, its cars had run a cumulative mileage of over 30,000,000 km [25]. Industry advocates’ ideal of ‘user can rent a car wherever he needs; sharing can replace buying’ will cost huge resources [4–6]. Even the monarchs of automobile industry such as BMW and Benz, cannot afford such ceaseless, huge expending of car-sharing service system. Car-sharing companies in China such as UUshare, Leshare, Ezzy, Car2go (Chongqing, China), and Togo closed their business in a short time owing to low utilisation and high investment problems. Fig 1 depicts the development process of Togo, a car-sharing company in China. Togo gained 6 rounds financing and over 70 million dollars in 3 years. With this money, Togo’s car-sharing business expanded to seven cities., But Togo closed its business in only 2 months after getting the B2 round financing due to inadequate profitability.

In book of *Competitive Strategy*, Michael Porter described ‘focus strategy’ [26]. When companies are unable to service the whole market, they can focus on a specific market segment or niche. A niche market is usually not large, but can be well served by company with limited resources. Accordingly, focus strategy can efficiently help car-sharing company tread through the high-cost and low-return crisis in the early stage. ‘Niche’ also means being advantageous in some market segments. Niche strategy would help car-sharing companies build a valuable image in the city transit market. Therefore, determining a niche market for car sharing is worth studying.

Because of its sharing attribute, niche market of car sharing could probably be drawn from special travel scenarios rather than special people groups. In the work ‘When is ours better than mine’, Lamberton and Rose believed that common people have two travel patterns: commuter/regular travel, with high demand and travel during workdays, and irregular travel, with low demand and occasional travel, such as meeting friends, travel in leisure time, and so on [27]. Considering the predictability and high frequency of usage, people prefer private transport for personal or individual use, such as private car, for the first pattern. For the second pattern, sharing rides is economical owing to the low demand for travel. Commercial sharing systems help combine infrequent consumption demands and supply centrally, thereby making it economical and profitable [28].

Although it is critical for car-sharing industry to work out the potential relationship between niche markets and travel scenarios, few studies have verified travel scenarios with real car sharing records. To be more exact, figuring out the best travel scenario under which car sharing is more beneficial than other transport pattern to meet people’s occasional travel demands is still a problem remaining to be solved. Accordingly, this paper develop a car sharing travel scenarios mining method with city’s POI data and car sharing records data. We imply this method in a case of car sharing program in Beijing, China. And further examine spatial-temporal characteristics and economic benefits of car sharing in the mined travel scenarios. These special travel scenarios should be determined as the niche market for the car-sharing service to help it tread through the crisis in the early stage of development.

## Literature review

### Typical user group and travel distance of car sharing

Sefage, the first car-sharing project, was introduced in Zurich, Switzerland in 1948. This project explores timesharing mode to meet people’s travel needs while avoiding the burden of owning a car. The concept of timeshare travel has gradually emerged in Switzerland; accordingly, Cooperative ATG and ShareCom, two large car-sharing organizations, were formed in the 1980s. The timesharing mode expanded in Europe and was introduced to the United States, Canada, China, and other countries in the 1990s.

Numerous studies have identified the typical user group of car sharing. Millard-Ball et al. determined that most users are aged 30 to 40 years and possess a higher education level [29]. Burkhardt et al. claimed that users are aged between 25 and 35 years, family size is 2.02, more than half of user’s annual income is above 60000$, and 72% users do not own a private car [30]. In their research in Seoul, Kim et al. claimed that major users are males aged between 20 and 30 years, own a car, are single, and belong to the white-collar group [31]. Hui et al. analyzed the information about registered members of Hangzhou and determined that 86% of users are males, 67% of users own cars, and 53.3% of them have more than a year of driving experience [32].

Moreover, some studies have discussed a suitable travel distance for car-sharing services. Hui et al. analyzed trade records and determined that travel distance is mainly concentrated around 30 to 50 km, indicating that under this travel range, the cost of taxi is much higher than that of car sharing [33]. Litman believed that car sharing may benefit people who travel less than 10,000 km a year [34]. Shaheen and Cohen believed that car-sharing users are motorists who drive 10000 to 16000 km per year [4].

However, several contradictions exist concerning user group and travel distance. Because car-sharing orders are based on users’ occasional travel demands, which makes it difficult to identify frequent customers. These contradictions can support our hypothesis that niche market for car sharing does not depend on user groups but travel scenarios.

### Travel scenarios in car sharing studies

Lamberton and rose believed and gave evidences that people with infrequent and irregular travel behaviour prefer car sharing than owning [27]. Kim et al. believed that car sharing can be used for occasional travel around the city centre. Because city center is short of parking, commuters usually go there by public transport [31]. When they are occasionally in emergency, driving car sharing can greatly saves their time. At the meantime, Kim et al. believed that car sharing is better for non-commuting trips such as going for entertainment after work [31]. Hui et al. considered the price advantage of sharing cars versus taxis and deduced that car sharing can be used for long-distance travel to multiple destinations [33]. Ciari et al. found that car sharing site near the residence has a greater impact on the users’ willingness than car sharing site near the work place, which indicates that car sharing is mainly used for non-commute activities such as shopping, meeting friends [35].

However, these studies on travel scenarios for car sharing were based on customer willingness survey or qualitative analysis, and were not supported by travel data. Existing quantitive studies based on travel data for car sharing only analyzed simple statistical features of travel distance, travel hour, or users’ demographic attributes without an in-depth investigation of travel scenarios. On that basis, our research further focusing on mining the travel scenarios with real car sharing records.

### Travel scenarios mining analyses

Many studies agreed that geographic information of the visited area, such as land use information, will help identify the travel purpose and detect some special travel scenarios [36, 37]. For example, Wang used point of interest (POI) data to define trip destinations and detect the relation between people’s mobile Internet usage behaviour and their preferred destinations [37]. Liu et al. used POI data and taxi origin and destination (OD) data to determine the function of city region [38]. Wang et al. determined eight spatiotemporal patterns of taxi trajectory: morning rush, daytime, evening rush, and night patterns on weekdays and weekends separately [39]. However, using geographic information to analyze car-sharing trajectory and travel patterns is rare in existing literature.

### Summary

Existing literatures provide important references for our research, suggesting that the target market of car sharing emerges from non-commuting trips, which are probably some infrequent and irregular travel scenarios. However, most of the existing studies have discussed car sharing travel scenarios by customer willingness survey or qualitative analysis methods. For example, going for entertainment after work, shopping, meeting friends and so on. But none of these scenarios was examined by real car sharing case. Meanwhile, many studies have used mobile phone traces data and city’s POI data to analyze people travel flows or predict people’s travel preference. We see the potential to explore car sharing travel scenarios with car sharing traces and land usage data.

This study uses electric car sharing records data and city’s land usage data to identify the representative travel scenarios for car sharing and provides positioning and target market selection suggestions for car-sharing services. The rest of this paper is organized as follows. First, this study introduces data used. Next, the study explains the research method. Thereafter, the results are presented. Finally, the paper presents conclusions, discusses the usefulness and limitations of the research, and provides directions for future research.

## Data

### Car-sharing data

Our study analyses travel data of a car-sharing company in Beijing, China. This company was founded in Dec. 2016, and its car-sharing app was launched in Nov. 2016. Its main shareholder is the largest local motor manufacture, who are trying to explore the commercial sharing model for future business and intents to promote electronic cars for motor manufactures.

The company provides free-floating electric car sharing service in Beijing which has the largest population and with highest car ownership in China. Beijing has executed an exceedingly strict traffic restriction since 2009 to ease traffic pressure. Private cars are divided into five groups according to a car’s tail number, and each group is prohibited to travel within the fifth ring road once a week. Electric cars do not count. Moreover, Beijing has executed another car-purchasing limitation policy. Every month, the government releases 7,500 new car licenses and randomly allocates them to applicants from a 2.8 million waiting pool. Thus, only a small proportion of people with driving license can own a private car. These policies create a huge gap of driving demands in Beijing and create opportunities for car-sharing company.

Data samples obtained were from 1 May 2017 to 30 May 2017, 6 months after the programme started. During this one-month period, the car-sharing platform operated 103 car fleets, and total records were 10560. Average records per vehicle per day is 3.41. The maximum daily rental record of a vehicle was 7. Each car-sharing record contained eight variables: order ID, lending time, returning time, rental fee, origin longitude, origin latitude, destination longitude, and destination latitude. When a user visits several places during a trip, only the OD locations are recorded. Stop locations during the trip cannot be observed by the data records.

### POI data

POI data is a pair of coordinates associated with the descriptions of the location, such as name, telephone, and categories of business. All human activities spots in the city, regardless of size, large places, like park, hospital, and university; small places, like convenience store, gas station, and public toilets, have their POI records. The POI data used in our study were extracted from Baidu Map Service 10.18.0. Baidu Map enables the registered developers to obtain a city’s POI data from the application program interface. In Beijing, our target area, approximately 386000 POIs of 20 predefined categories can be obtained. We deleted eight categories of POIs records: ‘Car maintenance’, ‘Motorcycle maintenance’, ‘Car sales’, ‘Car service’, ‘Place name and address’, ‘Road ancillary facilities’, ‘Transport service’, ‘Communal facilities: toilet and telephone’. Because these records were related more to map navigation information rather than location property information. We retained 12 categories of POIs records to define the area function: ‘Restaurant’, ‘Tourism’, ‘Company’, ‘Shopping service’, ‘Finance and insurance’, ‘Education’, ‘Residence’, ‘Life and community service’, ‘Sports, leisure and entertainment facilities’, ‘Health care service’, ‘Government and public service organization’, ‘Hotel’. For example, a 1km^2^ city grid cell near campus city in the northwest corner of Beijing, the location coordinate of the grid’s center is (116.25°E, 40.24°N). It contains 4024 spots of POI records. After deleting spots belong to eight navigation categories. It leaves 826 spots, including 77 spots of ‘Restaurant’, 0 spot of ‘Tourism’, 13 spots of ‘Company’, 242 spots of ‘Shopping service’, 22 spots of ‘Finance and insurance’, 34 spots of ‘Education’, 97 spots of ‘Residence’, 107 spots of ‘Life and community service’, 25 spots of ‘Sports, leisure and entertainment facilities’, 42 spots of ‘Health care service’, 54 spots of ‘Government and public service organization’, and 113 spots of ‘Hotel’.

## Methodology

**Fig 2:**
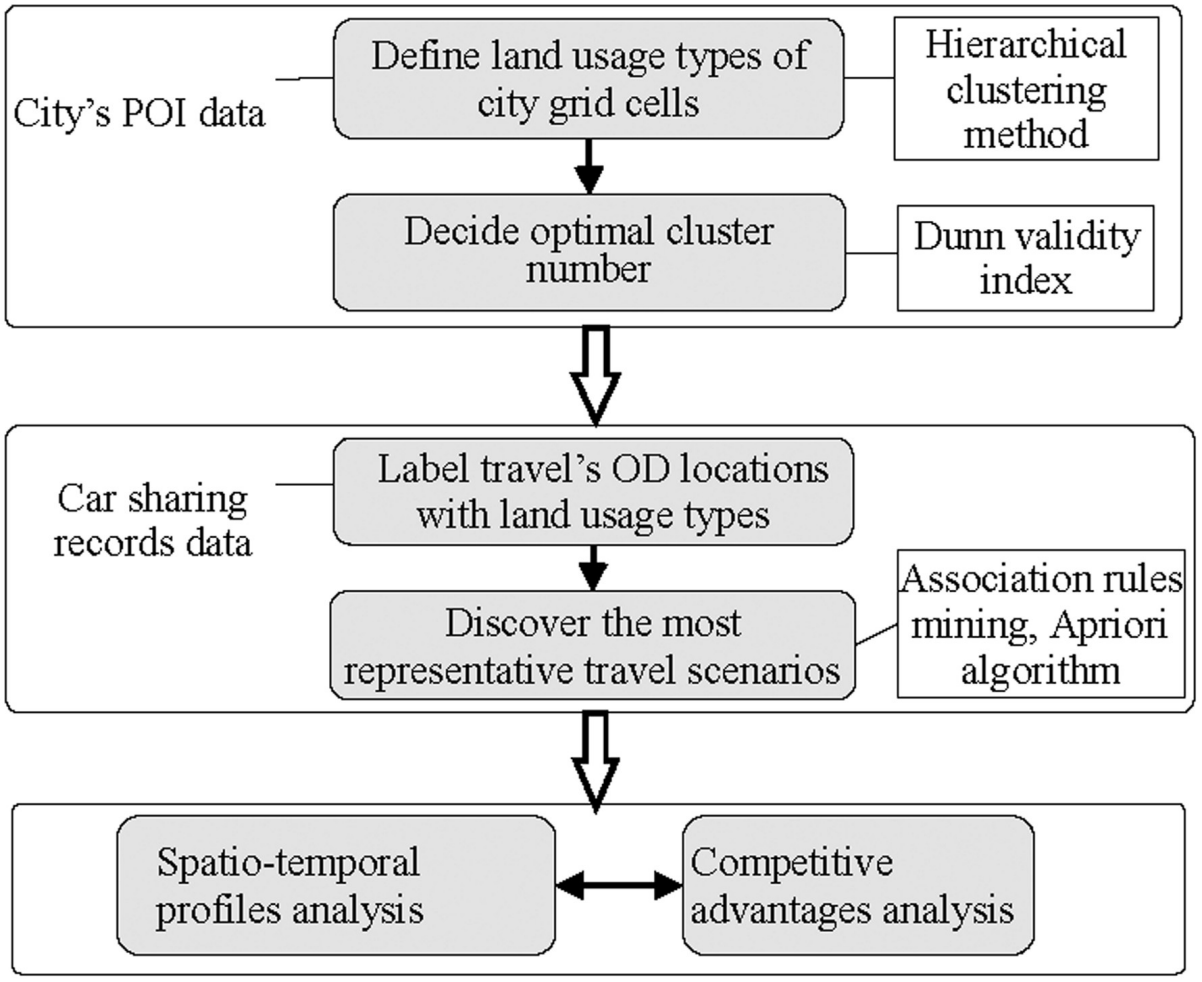
Flowchart of research method.

In Fig 2, we present a flowchart of the proposed research method in this study. First, land usage types of city’s grid cells are defined by hierarchical clustering method based on city’s POI data. Dunn validity index is used to examine the optimal cluster number. Second, OD locations of each car-sharing trip can be labelled by the land usage types. Third, typical travel scenarios are extracted by analyzing association rules of these OD land usage type pairs, and users’ travel preferences can be observed from rules appearing more frequently in car sharing trip records. In the end, spatio-temporal patterns and competitive advantages of car sharing in the typical travel scenarios are tested.

### Land usage types of city’s grid cells

**Fig 3:**
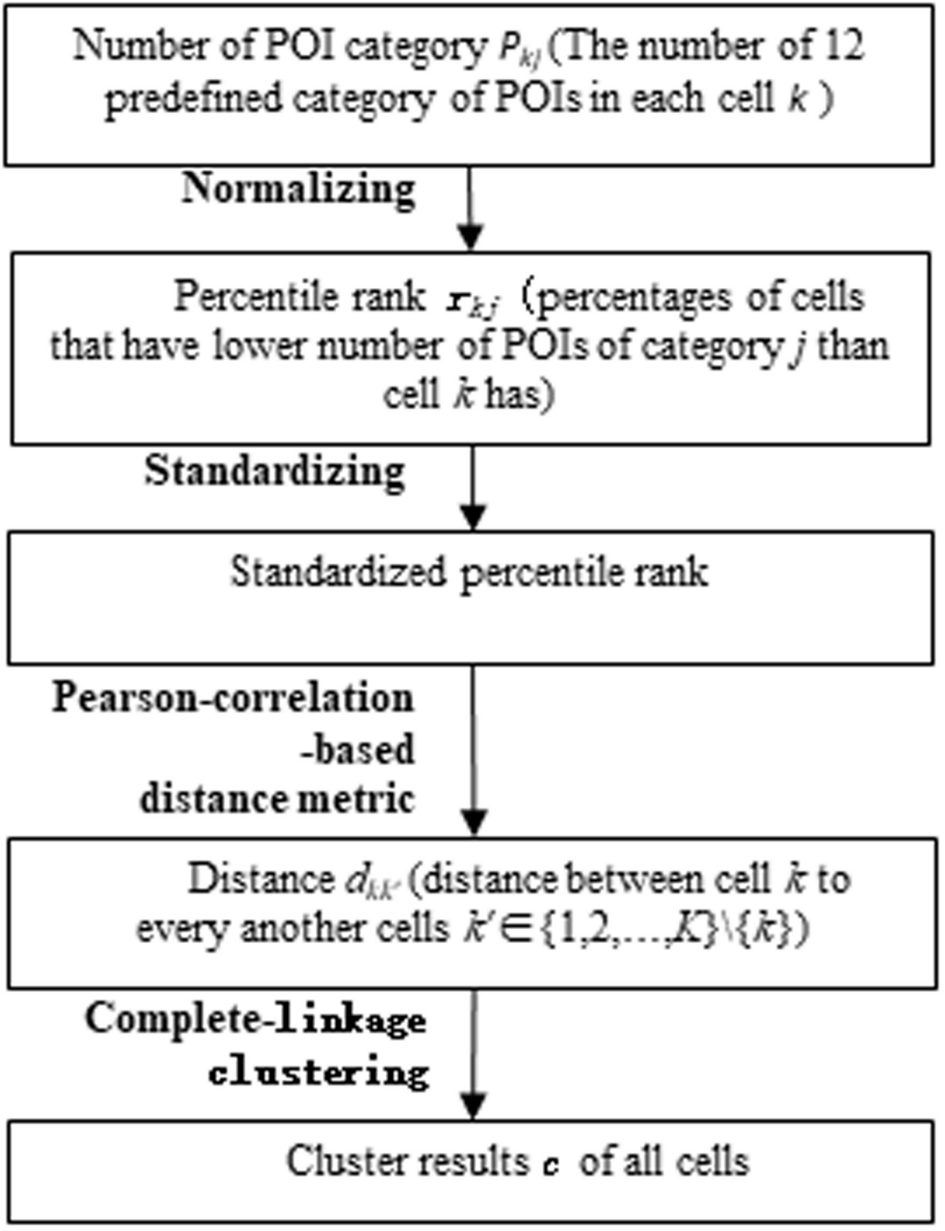
Flowchart of the hierarchical clustering method (each grid cell size 1 km × 1 km, *k* →cell, *j* →POI category).

Land usage types of OD locations can determine travel demand in traditional models [40–43]. For example, if Origin (O) location is ‘residence’ and Destination (D) location is ‘catering or shopping centre’, we can identify the trip as a going for entertainment travel. One solution is to distinguish the OD locations types with geographical information of area land use [41, 44]. Many scholars have selected POI data as the best data resource, which contains more detailed land usage information in the city [36, 45–47]. Therefore, the land usage information with POI data was used to cluster city’s grid cells. Clustering results will serve as a label to describe the place function of OD locations. Fig 3 presents the flowchart of the clustering method.

First, the city is divided by a virtual grid coordinate system [46, 48]. For a cell *k*∈{1,2,…,*K*}, the number of each predefined category of POIs is calculated, named *P*_*kj*_, where *j*∈{1,2,…,*J*} denotes a POI category (e.g., restaurant or workplace). The number of POIs of each category is then ranked over all cells, and the percentile rank *r*_*kj*_ is calculated as the percentages of cells with lower number of POIs of category *j* than cell *k* has. Accordingly, each cell *k* can be described as a vector of the percentile ranks of all the POI categories ***r***_***k***_ = (*r*_*k*1_,*r*_*k*2_,…,*r*_*kJ*_).

Second, assume that the similarity between the types of two grid cells can be reflected by the correlation between vector ***r***_***k***_ and vector ***r***_***k***′_, where *k*′∈{1,2,…,*K*}\{*k*}. Because correlation is scale-invariant, it is better to standardize ***r***_***k***_ as ${\hat{r}}_{k}$ to represent the profile of a cell, whose element is calculated as follows:

$${\hat{r}}_{kj}=\frac{r_{kj}-{\bar{r}}_{k}}{\sqrt{\sum_{j\in J}{\left(r_{kj}-{\bar{r}}_{k}\right)}^{2}}}$$ (1)

where ${\bar{r}}_{k}$ denotes the mean of ***r***_***k***_.

Third, Pearson-correlation-based distance metric can help calculate the distance *d*_*kk*′_ between these two vectors [49, 50], as in the following equation:

dkk′=1−cov(rk∧,rk′∧)s(rk∧)s(rk′∧) (2)

where *cov* (${\hat{r}}_{k}$, ${\hat{r}}_{k^{\prime }}$) denotes the covariance of ${\hat{r}}_{k}$ and ${\hat{r}}_{k^{\prime }}$; *s* (${\hat{r}}_{k}$) denotes the standard deviation of ${\hat{r}}_{k}$; and *s* (${\hat{r}}_{k^{\prime }}$) denotes the standard deviation of ${\hat{r}}_{k^{\prime }}$.

Finally, the complete-linkage clustering method can help identify relatively more compact clusters of approximately equal diameters [51, 52]. Thereafter, the clustering results of each cell’s representative land usage are generated, named land type *c*.

### Dunn validity index analysis

Consider that the land type *c∈* {1, 2, …, *C*} of each grid cell can be sensitively changed using some clustering parameters. One of the most important parameters is the total number of clusters. We used Dunn validity index (DVI) to select the optimal clustering number, *C*. DVI is an evaluation index for clustering algorithm [53]. In the process of clustering, each cluster’s compactness will be calculated using the DVI, defined as the ratio of the smallest distance between observations in different clusters to the largest intra-cluster distance, as follows:

$$DVI=\frac{\underset{0<m\neq n<K}{\text{min}}\{\underset{\forall {\overset{\wedge }{r}}_{k}\in \Omega_{m},\forall {\overset{\wedge }{r}}_{l}\in \Omega_{n}}{\text{min}}\{\left\|{\overset{\wedge }{r}}_{k}-{\overset{\wedge }{r}}_{l}\right\|\}\}}{\underset{0<m\leq K}{\text{max}}\;\underset{\forall {\overset{\wedge }{r}}_{k},{\overset{\wedge }{r}}_{l}\in \Omega_{m}}{\text{max}}\{\left\|{\overset{\wedge }{r}}_{k}-{\overset{\wedge }{r}}_{l}\right\|\}}$$ (3)

Where *${\hat{r}}_{k}$ and ${\hat{r}}_{l}$* denote the standardized POI vectors of two different cells *k* and *l* in the city; *m* and *n* denote different cluster groups. ${\hat{r}}_{k}\in \Omega_{m}$, *${\hat{r}}_{l}\in \Omega_{n}$* mean the two cells are classified into different cluster types, and ${\hat{r}}_{k}$, ${\hat{r}}_{l}\in \Omega_{m}$ mean the same cluster type.

Maximum DVI means the optimal clustering number, and the most distinctive clusters are obtained. The distance used to calculate the DVI is the Pearson-correlation-based distance defined previously.

### Association rule mining of OD land usage type pairs

Association rule mining is well-suited for discovering a particular travel behavior, since the mined ‘if–then patterns’ represent high frequent trips, without knowing the specific features for travel behaviors (when to travel or utilization times per day). This approach was initially used to analyze market basket data and was gradually applied in many areas. In recent years, many scholars have used association rules to research travel behavior data. Lu proposed the methodology to identify daily operational incidents in Shanghai Metro network [54]. Zhao et al. developed a new association rule algorithm to analyze bus-bicycle transfer behavior using metro and public bicycle data [55]. Guo et al. used the association rule to identify commuting travel mode using Beijing Subway data [56]. No previous studies have examined car-sharing travel behaviors using data mining.

Association rule mining can discover the interest linkage patterns, such as identifying people in location A who are more likely to go to location B. In general, we can say that if A has a great possibility (larger than a fix threshold) to move to B, then we get the rule, A→B. The cluster types of OD locations are two-value dataset as shown in Table 1. Using association rule mining, we can get support, confidence, and lift rates of each mode, like *c*_*i*_ → *c*_*j*_, in more detail. Support rate means how much historical data supports the rule, calculated as the joint probability of *c*_*i*_ and *c*_*j*_. Among rows containing *c*_*i*_, and the equation is as follows:

$$Support\;\;(c_{i},c_{j})=P(c_{i}c_{j})=\frac{number\;(c_{i}c_{j})}{number\;(All\;Samples)}$$ (4)

**Table 1:** Examples of OD cluster type data.

Record Item	Cluster type of Origin (O)	Cluster type of Destination (D)
1	*c*_*1*_	*c*_*2*_
2	*c*_*1*_	*c*_*3*_
3	*c*_*2*_	*c*_*4*_
4	*c*_*2*_	*c*_*1*_
5	*c*_*1*_	*c*_*3*_
6	*c*_*4*_	*c*_*5*_
7	*c*_*5*_	*c*_*1*_
8	*c*_*1*_	*c*_*5*_
9	*c*_*1*_	*c*_*3*_
10	*c*_*2*_	*c*_*6*_

Confidence rate means how confident we are that the rule holds true. Confidence is the conditional probability of *c*_*j*_ given *c*_*i*_. It is calculated as follows:

$$Confidence\;\;(c_{i}\leftarrow c_{j})=P(c_{i}\left|c_{j}\right.)=P(c_{i}c_{j})/P(c_{j})$$ (5)

Lift rate is the ratio of confidence rate to support rate. If lift rate is < 1, then *c*_*i*_ and *c*_*j*_ are negatively correlated and otherwise positively correlated. If it is equal to 1, then *c*_*i*_ and *c*_*j*_ are not correlated.


$$Lift\;(c_{i}\leftarrow c_{j})=P(c_{i}\left|c_{j}\right.)/P(c_{i})=Confidence\;(c_{i}\leftarrow c_{j})/P(c_{i})$$ (6)

## Results and discussions

### Land usage types of city grid cells

This research choose Beijing new city center, which is mentioned in ‘2019 Beijing Urban Master Plan for the next 20 year’, as target area. This area includes six districts: Dongcheng, Xicheng, Haidian, Chaoyang, Shijingshan, and Fengtai. Following the method of Wang et al. [37], we divided the target area into 2841 grid cells of 1 km^2^.

**Fig 4:**
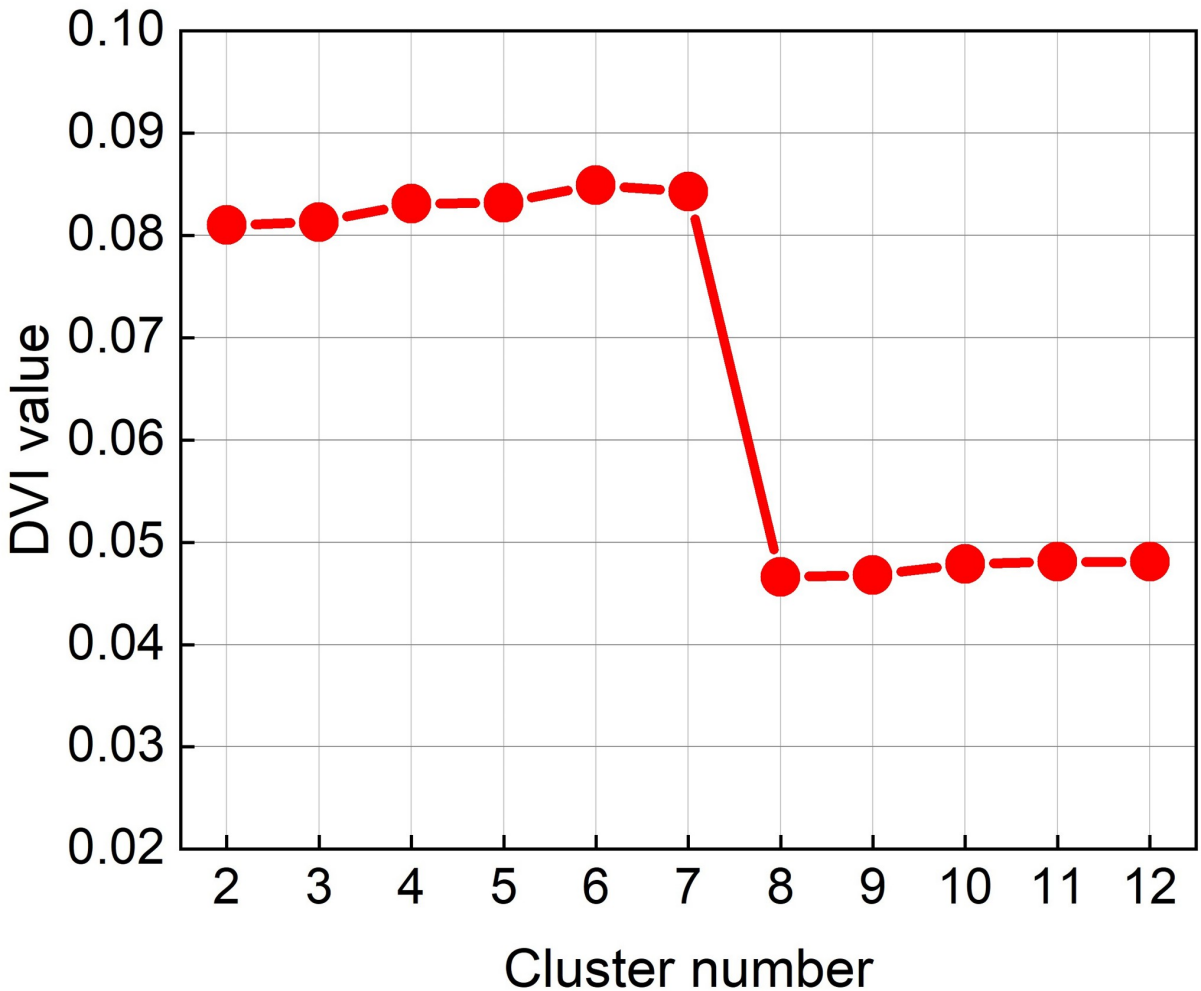
DVI used to determine the number of clusters.

**Fig 5:**
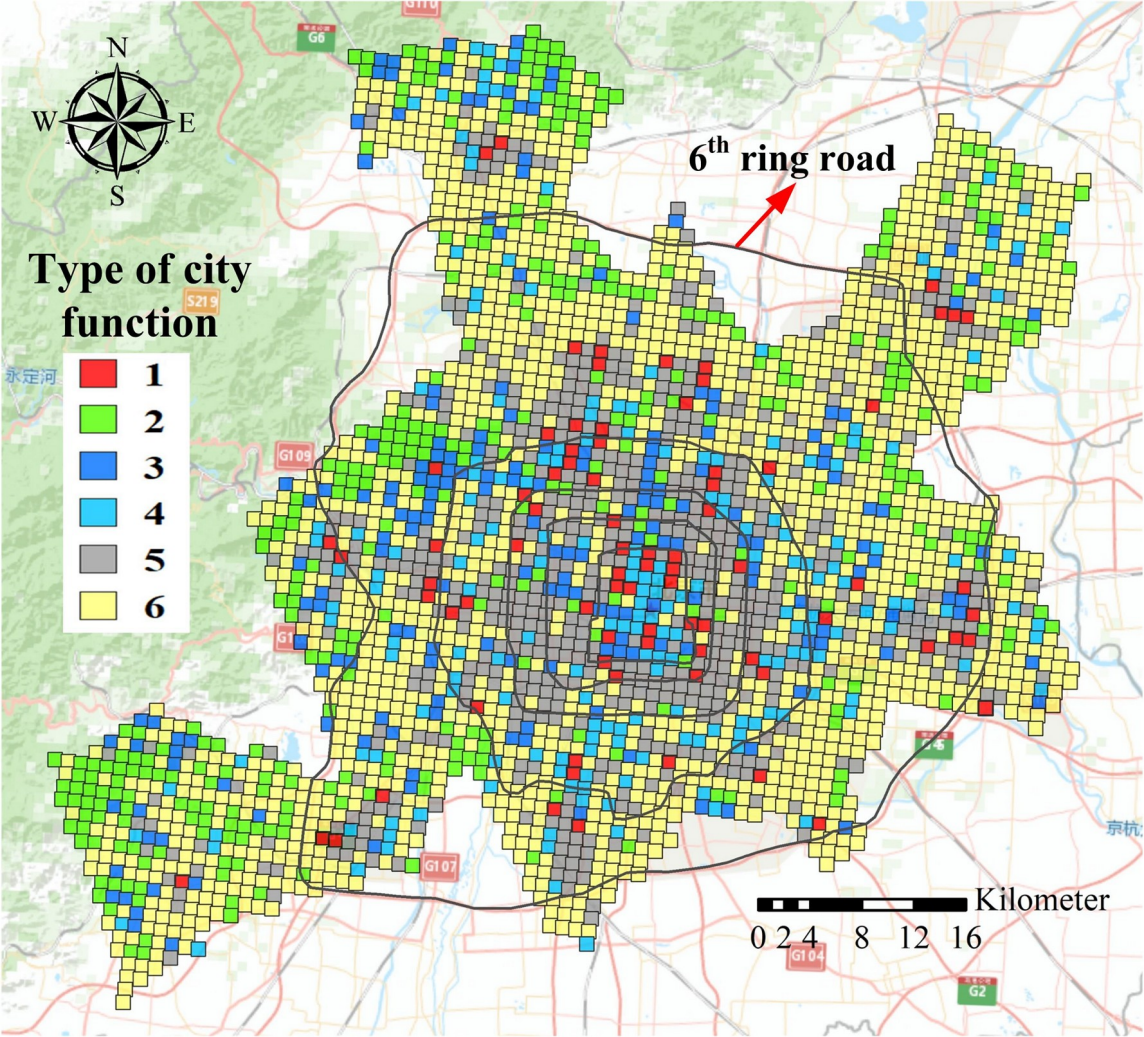
Clustered grid cells of target area in the city.

Clustering method explained in section *“Land usage types of city’s grid cells”* is used to define land usage types of city grid cells. For each parameter of clusters number, we got a vector of clustering results, and also a DVI index to value the effectiveness of this clustering. As shown in Fig 4, the parameter of clusters number is chosen from 2 to 12. 12 is the number of business categories of POI samples. DVI index climbs slowly to the top at 6 and 7, then sharply falls to a lower level. That means the clusters are best distinguished by setting the clusters number as 6 or 7. The smaller number 6 is chosen for simplicity. Fig 5 shows clustering results of this area.

**Fig 6:**
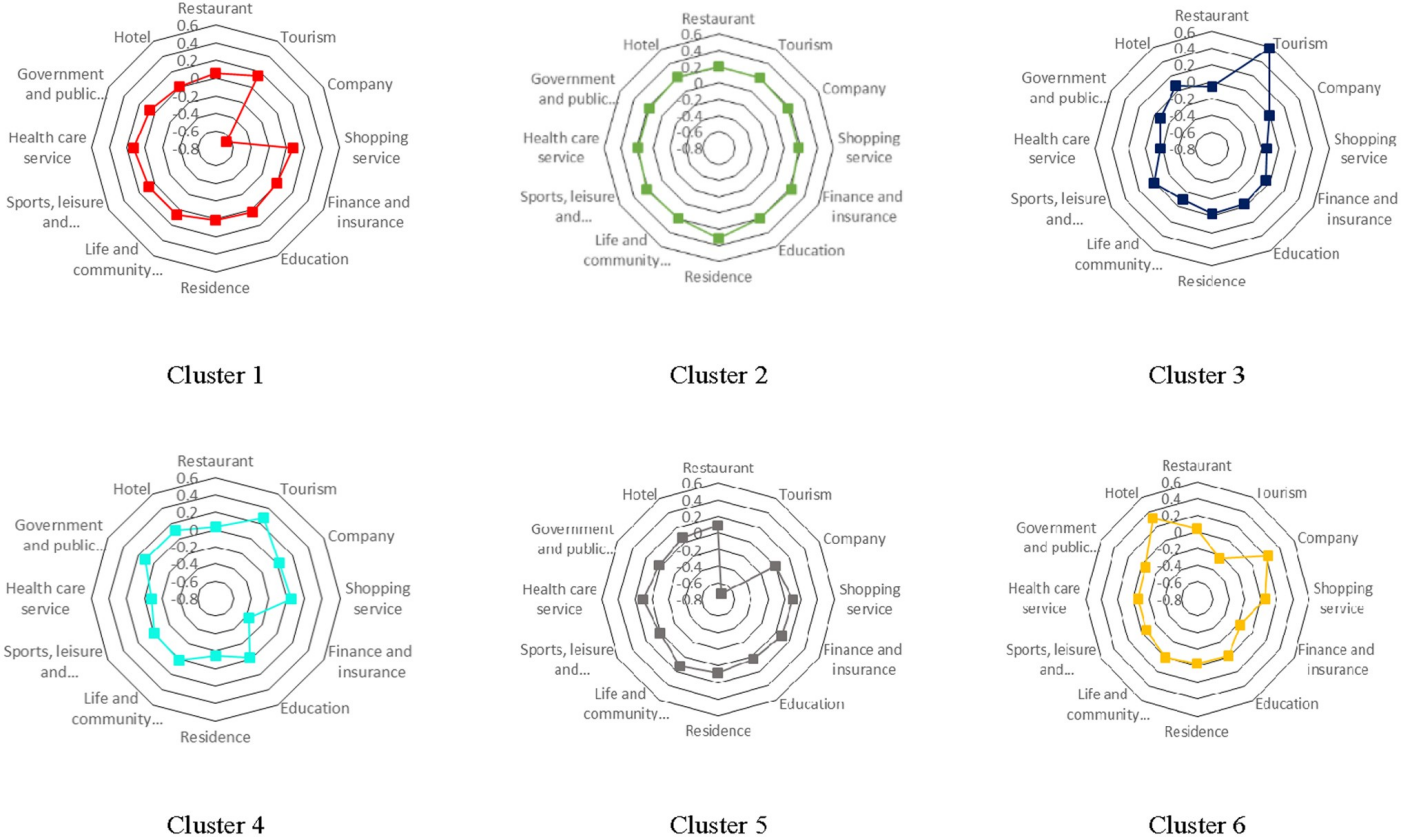
Radar chart of each cluster.

Table 2 shows the profile of each cluster type, where typical cases are famous spots in this type of cells. Fig 6 gives the radar chart of each cluster. In radar charts, point in each axis is calculated as the average value of percentile rank *r*_*kj*_ of all cells belonging to cluster *k* (see Section *“Land usage types of city’s grid cells”* for the definition). A higher average value of *r*_*kj*_ means the POI category *j* is more influential. Thus, radar chart can indicate which POI categories are dominant in that cluster. The changeful shapes displayed by 6 radar charts mean that dominant POI categories of each cluster are quite distinct from others. Based on them we give the interpretation of each cluster type in the Description Column of Table 2.

**Table 2:** Profile of each city cluster type.

Cluster	Description	Typical cases
1	Residential area in old town, with complete property, mainly inside the city, a few stay in centres of suburban counties	People’s Publishing House second working area; Residential area of Tsinghua University; Lanxiyuan Community, Cangshang Community and Xingang Community in Shunyi District; Longtengyuan Community in Changping District.
2	Natural ecological areas with less human activities, include forest, water area of rivers and lakes, apron of airport,	Northern side of Fragrant; Wenyu river; Mangshan Hill; Wetland park of Yizhuang; Tonghui river; Beijing Capital International Airport.
3	Touristy spots, historic sites, and city parks	Purple Bamboo Park; Beijing Zoo park; Eight Great Temples of the Western Hills; Taoranting Park; Olympic Forest Park; Canal Cultural Plaza.
4	Mix of public service and industrial areas, inside the city	Harmonious Culture and Creative Industry Park; Banbidian Cultural Industrial Park; Xiaohongmen International Corporate Culture Park; Liangxiang Campus of Beijing Institute of Technology.
5	Commercial area, mostly inside the third ring road of the city	Peking University Third Hospital Capital Library of China International Trade Building Shouhui Healthy Techno Park
6	Suburban commercial and residential areas, mostly outside the third ring road of the city	The Capital Steel Corporation’s old site/ Chinese animation game City Shahe University Town China International Exhibition Center (New site) Fengtai Science and Technology Park

Tourism, Company, Finance & Insurance, and Residence, the four POI categories play the most important role in distinguishing the clusters. Clusters 1 and 3 represent areas with more tourist spots, but Cluster 1 has much fewer companies. Because Cluster 1 includes areas in the old town of Beijing, the place hosts famous historic sites and old residential communities. Thus, the relative importance of company is lower in Cluster 1. Tourism is the highest in Cluster 3, indicating that Cluster 3 predominantly includes tourist areas. Clusters 5 and 6 are commercial areas with more workplaces and less tourism. Cluster 5 includes cells within the city centre, and Cluster 6 includes cells outside the city centre. Most companies in Finance & Insurance industry are rich and prefer to locate in the city centre. Thus, the average *r*_*kj*_ of Finance & Insurance is higher in Cluster 5 than that in Cluster 6. Cluster 2 is special in terms of the relative importance of all POI categories. Cluster 2 represents natural ecological areas with less human activities. The indices of all POI categories rank near 0.2; thus, the relative importance of all POI categories remains same. The areas belonging to Cluster 4 seem more multifunctional. They are almost similar to the areas belonging to Cluster 6, except that Cluster 4 possesses a higher number of public services and lower number of hotels.

As a conclusion, six land usage cluster types are defined as 1) residential area in old town; 2) natural ecological areas with less human activities; 3) touristy spots, historic sites, and city parks; 4) mix of public service and industrial areas inside the city; 5) commercial area inside the city; 6) suburban commercial and residential areas.

### Representative car-sharing travel scenarios

Using methods in Section *“Association rule mining of OD land usage type pairs”*, and setting the threshold of support rate as 10%, setting confidence rate as 30%, seven most effective association rules are mined from OD land usage type pairs, as shown in Table 3.

**Table 3:** Representative OD types of car-sharing travel.

OD types pairs	Support rate (> 10%)	Confidence rate (> 30%)
**5→5**	16.9%	30.5%
**6→5**	14.3%	34.7%
**3→6**	12.9%	31.2%
**6→3**	12.9%	31.2%
**3→5**	12.4%	30.1%
**1→5**	6.4%	38%
**1→3**	6.0%	35.2%

Travel purposes of the seven most effective association rules can be sorted into 3 situations.

First, trips between commercial places in city centre. The largest association rule 5→5 constitutes 17% of the total car-sharing records. Considering that the travel range of 5→5 is commercial areas of the city centre, we can figure out a part of demand of 5→5 is ‘day-time business trips to multiple destinations’. According to previous interview done by our work group, day-time business trips contribute 60% orders of ‘Yidu’ car-sharing company. Users in this travel scenario need to stop at several destinations and take brochures, promotional products, gifts, and so on and thus prefer to rent a car without a driver for flexibility, convenience, and privacy. We define “5→5” trips as “trips between commercial places in city centre”.

Second, trips going to or leaving from Cluster 5, including 6→5, the trip from ‘commercial or residential place outside the city’ to ‘commercial place inside the city’; and 1→5, the trip from ‘residential area in old town’ to ‘commercial place inside the city’. Considering both Cluster 6 and Cluster 3 contain large proportion of residential areas, and Cluster 5 are commercial areas in city center, we define “go and leave Cluster 5” trips as “commuting trips”.

Third, trips going to or leaving from Cluster 3, including 3→5, the trip from ‘touristy spots, historic sites, and city parks’ to ‘commercial place inside the city’; 1→3, trips from ‘residential area in old town’ to ‘touristy spots, historic sites, and city parks’; 3→6 and 6→3, the round trips from ‘commercial or residential place outside the city’ to touristy spots, historic sites, and city parks’. These trip modes are typical and stand to reason because most public transport lines are designed to link the city centre with tourist spots. People residing outside the city cannot reach tourist spots without changing lines. Thus, driving sharing car between ‘residential place outside the city’ and ‘tourist spot’ became a brilliant choice. We define “go and leave Cluster 3” trips as “city short-distance tourism travels”.

### Spatiotemporal profiles and competitive advantages of car sharing in typical travel scenarios

#### Day-time business trips and evening entertainment trips in city centre

**Fig 7:**
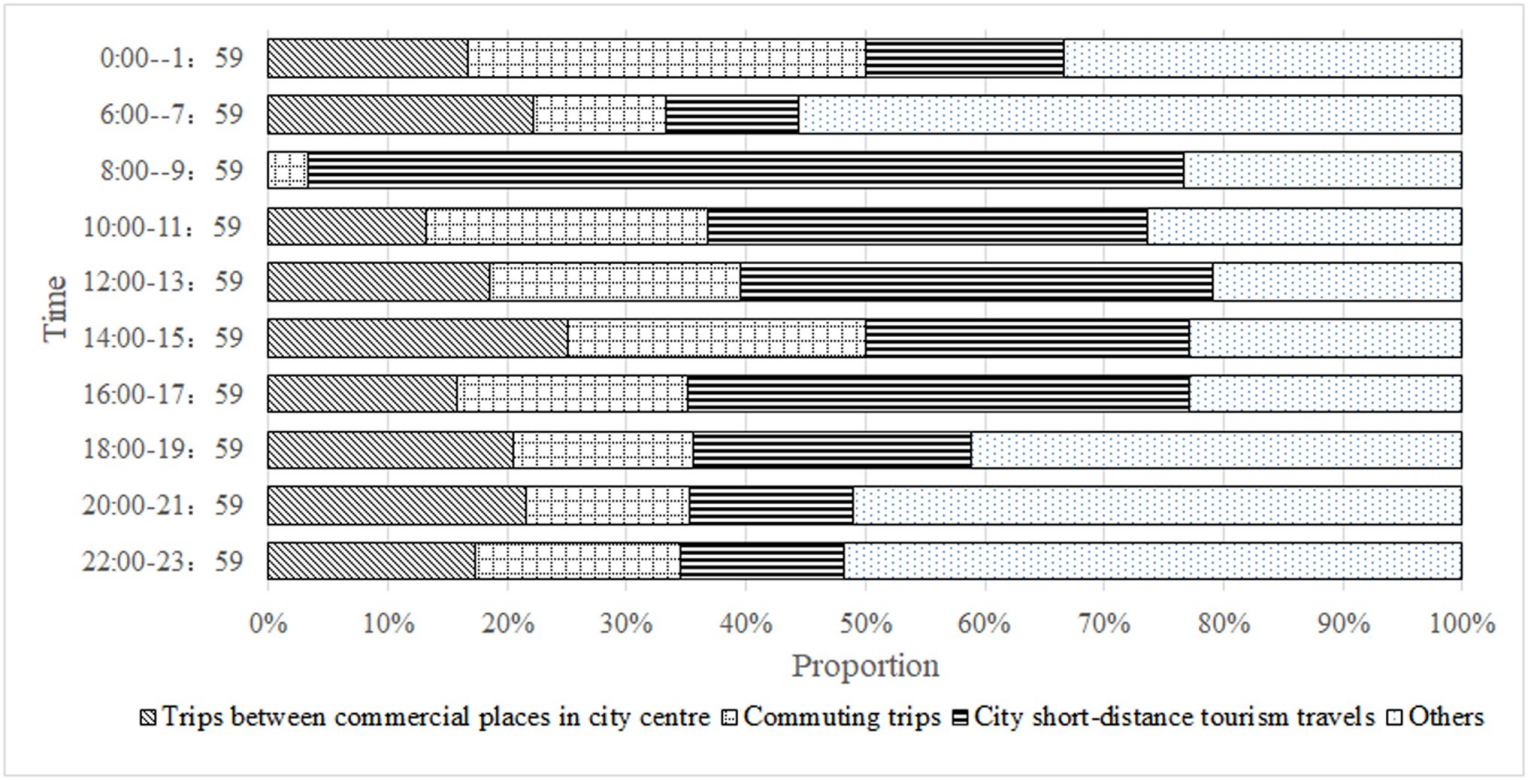
Proportion of typical car-sharing travel scenarios in every 2h records for a day.

Fig 7 presents the proportion of typical car-sharing travel scenarios in every 2 hours in a day, excluding dispatching and recharging time from 2:00–6:00. We can see the travel scenario of trips between commercial places in city centre mostly happened in daytime and evening, from 10 am to 11 pm. As mentioned above, a large part of demand is “day-time business trips to multiple destinations”. The other part of demand could be “travel for leisure and entertainment in the evening”.

#### Commuting trips in off-peak time

**Fig 8:**
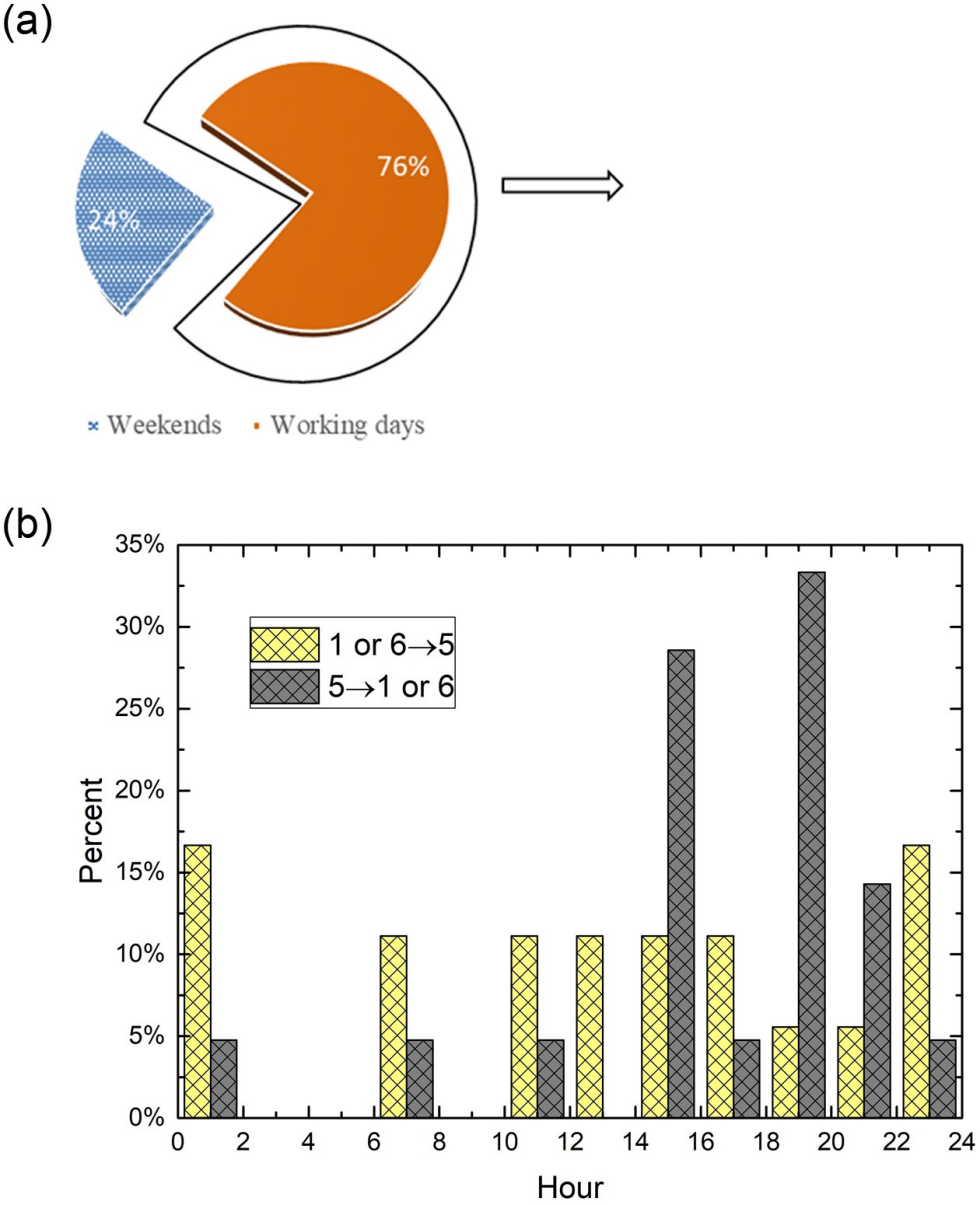
a Proportion of commuting trips in working days and weekends, b Commuting trips in every 2h records for working day.

The representative commuting travel scenarios are trips from old town and suburban areas to city centre’s commercial areas. Fig 7 show commuting trips occurred uniformly during the period from 10 am to 12 pm rather than morning-evening rush hours. Remarkably, the distribution ratio of commuting trips happened even less than other periods in the morning rush hours from 6 am to 10 am. In order to analyze this travel scenarios in depth, we calculate the commuting trips records in working days and in weekends separately, and draw the proportion of records in every 2h for working day. As shown in Fig 8a, commuting trips in weekends are much less than that in working days. It makes sense that people don’t go to commercial areas in the weekends or holidays. And because the records of commuting trips in weekends are scarce in our case, we only analyze the temporal distribution of commuting trips in working day. As shown in Fig 8b, trips of 1→5 and 6→5, which are travel demands from old town and suburban residential areas to city centre’s commercial areas, happens evenly throughout the day. Whereas trips of 5→1 and 5→6, which are travel demands from city centre’s commercial areas to old town and suburban residential areas, have two consumption booms, one occurs in 2pm-4pm in the afternoon, another occurs in 6pm-10pm in the evening. It is probably that the off-duty hours are less concentrated than start hours, and many people have some time to spare after works, so they can order a timeshare rental car and go back home. These results don’t mean that car sharing has become a useful communing tool. Instead of, car sharing can only use for communing trip during off-peak time.

#### City short-distance tourism travels

**Fig 9:**
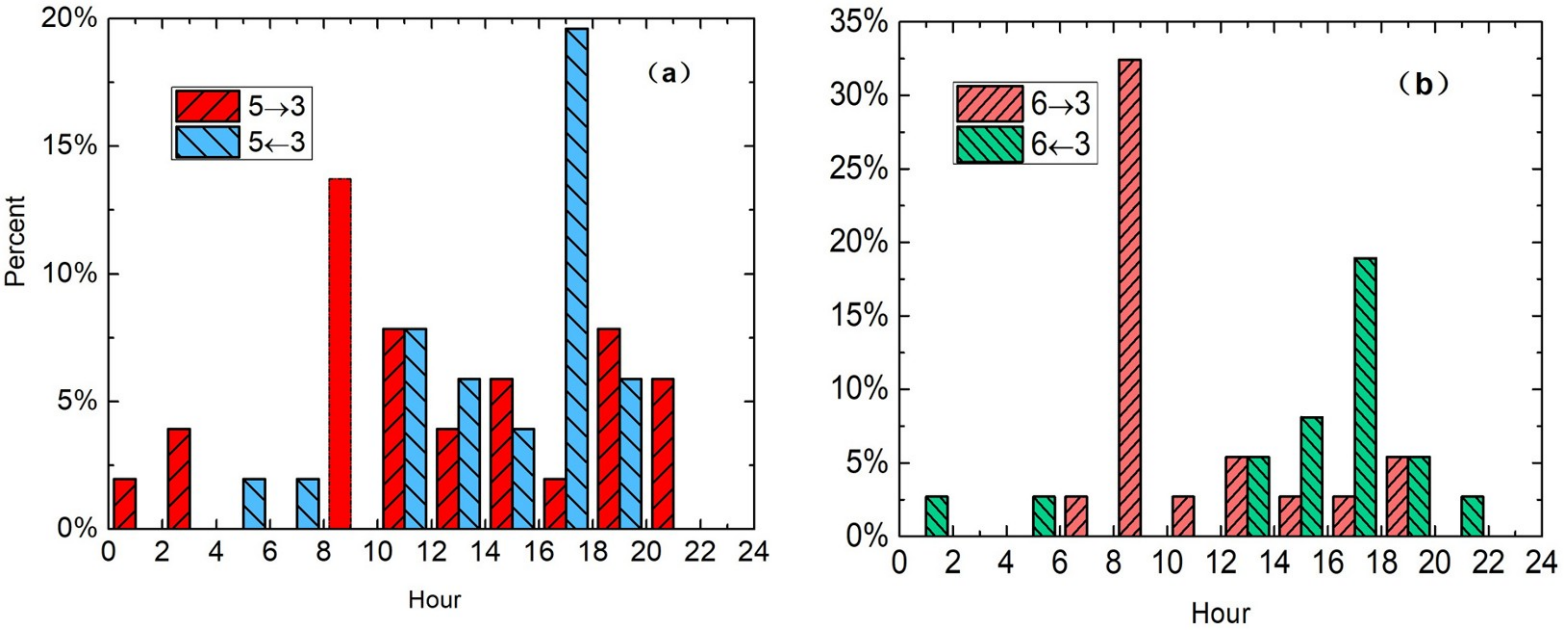
Temporal distribution of 4 representative tourism car-sharing travel scenarios.

Tourism travel scenarios constitute more than 1/3 of total car-sharing records. In Hainan Island, a popular seaside resort in China, ‘Xiao’er’ car-sharing company places their cars in scenic spots, hotels, and transport hubs and supports their users’ self-driving tour. ‘Yidu’ car-sharing company cooperates with Hanting hotel chains in Beijing to provide travel vehicle for their business guest and tourist guest. In Fig 7 we can observe city tourism travels have two demand peaks in a single day. Fig 9 shows the proportion of records in four scenarios separately. In the temporal distribution of 5→3 and 6→3, around 8am-10am in the morning, a period avoiding the morning rush hours, there are large demands for traveling to tourist spots; while in the temporal distribution of 5←3 and 6←3, in the afternoon, a demand hump emerges for leaving tourist spots. These leaving demands start to show in the midday and reach a peak before 6 pm. All these car-sharing records conform with city short-distance tourism travel that citizens go out a little late in the morning, come back a little early in the afternoon, and arrange their tourism travel to avoid the traffic rush hours.

#### Spatial features of typical travel scenarios

**Fig 10:**
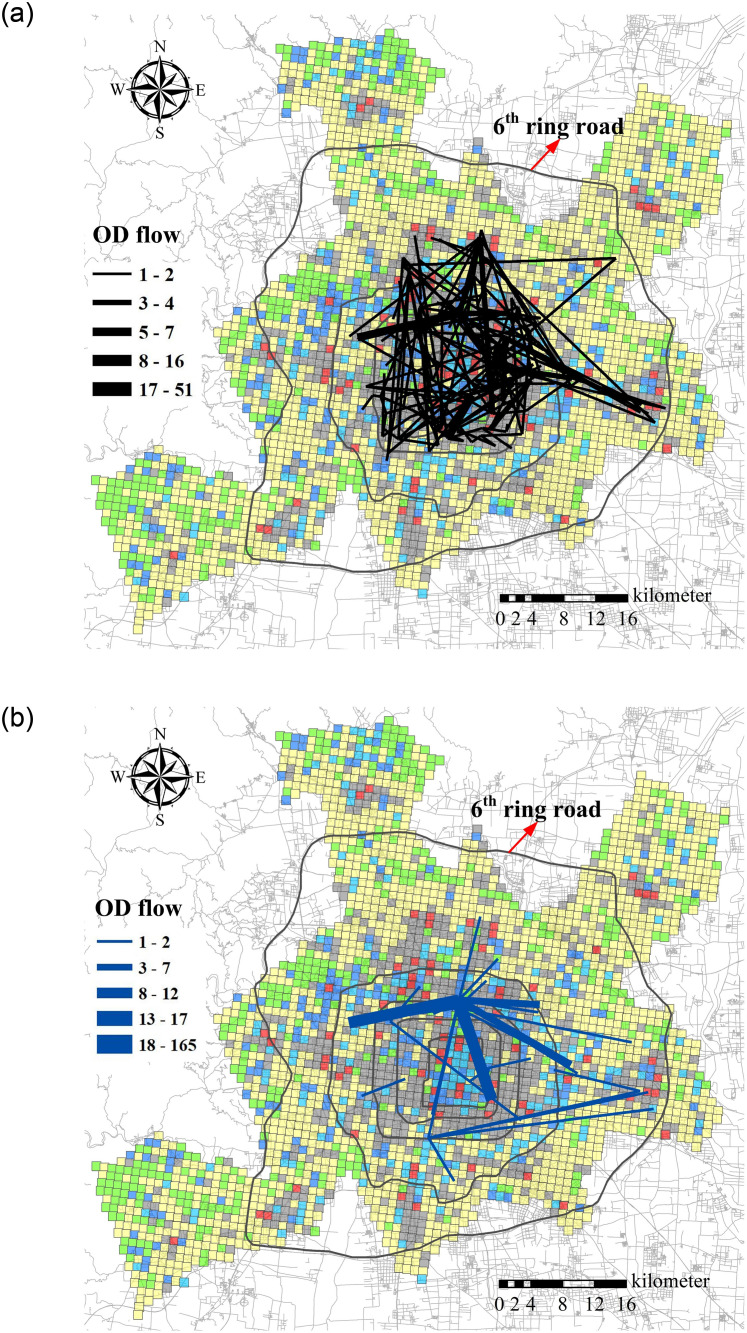
a OD flow of trips surrounding Cluster 5, b OD flow of trips surrounding Cluster 3.

Fig 10 shows the spatial features of the three representative travel scenarios trips in map. Travel scenarios of “Day-time business trips and evening entertainment trips in city centre” and “Commuting trips in off-peak time” are both related to Cluster 5, and are shown together in Fig 10a. Travel scenarios of “City short-distance tourism travels” are shown in Fig 10b. Comparing traces in these two maps, it is obvious that all travel scenarios trips have covered a large range of the city, even intrude into the regions within 5th ring road and 6th ring road. Moreover, almost all trips have a long travel distance. This may because user can drive the timeshared rental car by oneself, and enjoy better privacy comparing with other sharing traffic mode, like taxi or ride-hailing. Then user prefer to go further place when they chose this service.

The differences between travel scenarios trips in the two maps are that travel traces of trips surrounding Cluster 5 scatter over the region inside 5th ring road; while travel traces of trips surrounding Cluster 3 are less and clear, some travel trace with quite large OD flows. It is probable that tourism travel demand happens analogously, so each route can gather large flows. These travel trace can help car sharing to figure out some tourism route, since there are already so many travel demands happened.

#### Competitive advantages of car sharing in typical travel scenarios

Table 4 shows the records duration and rental fee of typical travel scenarios. By calculating all records belonging to each travel scenario, we find ‘Short-distance city travels for tourism’, has the longest renting time, around 4 h. The average renting time of ‘day-time business trips and evening entertainment trips within the city centre’ is 2.6 h. The average renting time of ‘commuting trips in off-peak time’ is 1.1 h.

**Table 4:** Comparisons of rental fee between car sharing and alternative traffic.

Description of travel scenarios	Record duration	Rental fee	Alternative traffic tool and fee	Car-sharing fee /Alternative traffic fee
Day-time business trips and evening entertainment trips within the city centre	2.6 h	46.8 YUAN	Taxi, 133.4 YUAN ^a^	35.1%
Commuting trips in off-peak time	1.1 h	19.8 YUAN	Traditional rental car, 107.3 YUAN ^b^	18.5%
Short-distance city travel for tourism	4 h	73.8 YUAN ^c^	Traditional rental car, 198 YUAN	37.3%

Notes:

^a^ According Beijing Taxi operation standard before 2018, if travel range within 3 km, charge 13 YUAN, if travel range larger than 3 km, the larger part will be charged 2.3 YUAN/km. An additional 20% fee will also be charged at nighttime, from 11 pm to 5 am. In 5–5, supposing drive 1.6 h, and take 1 h for customers visiting, and driving speed is 40 km/h, so the taxi fee is 133.4 YUAN.

^b^ Giving the daily rent fee of Chevrolet Cruz in Shenzhou car rental company for reference, the rental fee is 198 YUAN/day.

^c^ Car sharing service of this case is charged as 0.3YUAN/min.

Then we analyze the rental fee of the travel scenarios, and compare it with service charge of taxi and traditional rental car. We find that the cost of car sharing is much lower than that of other alternative modes and constitute only 20–40% cost of the latter, indicating that car sharing is beneficial over other modes in travel scenarios obtained in our research.

## Conclusions and recommendations

By analyzing travel data from a car-sharing company in Beijing, this paper indicates that three most representative travel scenarios of car-sharing service are ‘day-time business trips and evening leisure and entertainment trips within the city centre’, ‘commuting trips in off-peak time’ and ‘short-distance city travel for tourism’. Many of these observed relationships are interpretable. For example, ‘short-distance city travel for tourism’ usually lasts half a day. Renting behaviours avoid the morning and evening rush hours of commuting. ‘Day-time business trips and evening leisure and entertainment trips within the city centre’ usually lasts 2 to 3 h and mostly happens inside the third ring road of the city. ‘Commuting trips in off-peak time’ happens rarely in weekends or holidays. If people chose to use timeshare rental car for commuting, they don’t use it in morning rush hour. Commuting trip during off-peak time usually lasts around an hour.

As for the spatial feature of car sharing trips, people who choose timeshare rental car can enjoy better privacy than other sharing traffic modes, which leads to longer travel distance for all three travel scenarios trips. Moreover, in order to find the competitive advantages of car sharing, we compare rental fee in the typical travel scenarios. We find car sharing has huge advantageous in cost saving. The rental fee of car sharing only take 20–40% of other alternatives sharing traffic modes in these travel scenarios.

### Theoretical implications

In this paper, we proposed a data mining method of identifying car-sharing travel scenarios, with the combination of travel data and land usage data. Based on these travel scenarios, we can explore the purposes and spatiotemporal characters of car-sharing trips. Therefore, the second theoretical implication of this paper is giving spatiotemporal consumption portraits of car sharing. These consumption portraits can serve as more concrete interpretations of the queries that ‘how do car-sharing companies run their business?’ and ‘what are car sharing’s contribution to the whole city transport system?’ In the end, we prove that car sharing has cost-saving advantage over other alternative transport modes in some special travel scenarios. Car sharing is helpful, with superiorities of keeping customer privacy, offering better performance in terms of price ratio, higher freedom, and greater convenience in the three representative travel scenarios. These findings can further prove the rationality and necessity of car sharing.

### Managerial implications

The managerial implications of our findings includes: firstly, our research help car sharing companies to determine their position accurately based on user’s travel purposes, behaviors characteristics and travel needs. For the car-sharing companies, the representative travel scenarios can be used to predict users’ travel trace, predict the lending or returning demand, and then ameliorate the car-sharing companies’ operation management policies. In addition, these travel scenarios can help the car-sharing companies to identify target customers and their driving behaviors, cultivate their loyalty by providing accurate services, and tread through the high-investment and low-revenue crisis in the early stage. If a car-sharing companies can provide their services more appropriately, users will accept the new traffic mode more easily and enjoy more benefits from car-sharing service. Because car sharing has the ability to satisfy many fragmented and personalized travel demands in the city, a healthy car-sharing industry can also help the sustainable development of city’s transport system.

Secondly, the spatial features of typical travel scenarios can help car sharing companies to select better service modes. For the travel scenarios of ‘day-time business trips and evening leisure and entertainment trips within the city centre’ and ‘commuting trips in off-peak time’, their travel traces are scattered over the areas inside city’s 5^th^ ring road. It is better to use area coverage mode to serve randomly generated demand with free-floating cars. Whereas the travel traces of ‘short-distance city travels for tourism’ have relatively fixed routes with large OD flows, which are better to be served with fixed rental stations and vehicles.

Finally, the temporal distribution of each travel scenarios can help car sharing companies to optimize their demand forecasting and vehicle scheduling policies. And our findings about competitive advantages suggest that car sharing companies should further improve their promotion policy in terms of cost-saving and privacy protecting.

### Limitations

Although we conducted this research using quantitive method based on data extracted from real comsumption situation, there still exist some problem which call for future improvements. As for data quality, what we have obtained is only one-month travel records pertaining to the car-sharing company’s operation, although it is the best available data we can get at present, it is somehow limited for a comprehensive empirical study. Even so, these representative travel scenarios found in this study are undoubtedly valuable business opportunities for companies in the initial emerging markets. In addition, city features and landscapes would somehow positively affect the market volume depending on these representative travel scenarios, and the operational material of our research came from car-sharing data in Beijing, a city which is not only the political and economic centre of China but also the ancient cultural capital of six dynasties, there are beautiful scenery and historic sites all over the city. All these characters lead to numerous commuting demands, business visiting demands, and tourism demands.

Besides, we only study electric car-sharing system. Electric car-sharing service is different from conventional fossil fuel-based car-sharing service due to the nature of charging requirement and mileage limitation of the vehicles. All the results of this paper might be used to explain electric car-sharing usage behavior and help the operation of electric car-sharing mode, but whether our conclusions are still workable for conventional car-sharing service is still need for further empirical practice.

Despite these research limitations, the significant and interpretable travel scenarios found in our research reveal the potentiality of using land usage data and car-sharing record data to enhance the explanatory travel behaviour in future research. Moreover, the car-sharing business analyzed in our research is free-floating mode, which can reflect customers’ free wills more effectively. Free-floating car-sharing company who are planing to penetrating car-sharing projects into the city or station-based company who have problems selecting the rental sites in the city can adopt the suggestion given in this study.

## References

[1] Fournier G, Seign R, & Goehlich V, et al. Carsharing with electric vehicles: a contribution to sustainable mobility? Interdisciplinary Management Research. 2015; 11:955–975.

[2] Kim K. Can car sharing meet the mobility needs for the low-income neighborhoods? Lessons from carsharing usage patterns in New York City. Transport. Res. Part A. 2015; 77:249–260.

[3] Amirkiaee S. Y., & Evangelopoulos N. Why do people rideshare? an experimental study. Transportation Research Part F. 2018; 55:9–24.

[4] Shaheen S. & Cohen A. Growth in worldwide carsharing: An international comparison. Transportation Research Record: Journal of the Transportation Research Board. 2007; 1992:81–89.

[5] Cervero R., Golub A., & Nee B. City car share: Longer-term travel demand and car ownership impacts. Transportation Research Record: Journal of the Transportation Research Board. 2007; 1992:70–80.

[6] Firnkorn J., & Müller M. What will be the environmental effects of new free-floating car-sharing systems? The case of car2go in Ulm. Ecol. Econ. 2011; 70:1519–1528.

[7] Elona P., Veronique V. A., & Dorina P. Cars as a status symbol: youth attitudes toward sustainable transport in a post-socialist city. Transportation Research Part F: Traffic Psychology & Behaviour. 2018; 58:210–227.

[8] Xue Y., Zhang Y., and Chen Y. An evaluation framework for the planning of electric car-sharing systems: A combination model of AHP-CBA-VD. Sustainability. 2019; 11:5627.

[9] Chun Y., Matsumoto M., Tahara K., Chinen K., & Endo H. Exploring factors affecting car sharing use intention in the Southeast-Asia region: A case study in Java, Indonesia. Sustainability. 2019; 11:5103.

[10] Millard-Ball A., Murray G., & Schure J T. Car-sharing as a parking management strategy. In Proceedings of the 85th Annual Meeting of the Transportation Research Board. 2006; Washington, DC, USA.

[11] Martin E., Shaheen S., & Lidicker J. Impact of carsharing on household vehicle holdings: Results from North American shared-use vehicle survey. Transportation Research Record Journal of the Transportation Research Board. 2010; 2143:150–158.

[12] Efthymiou D., Antoniou C., & Waddell P. Factors affecting the adoption of vehicle sharing systems by young drivers. Transp. Policy. 2013; 29:64–73.

[13] Alfian G., Rhee J., Kang Y., & Yoon B. Performance comparison of reservation based and instant access one-way car sharing service through discrete event simulation, Sustainability. 2015; 7:12465–12489.

[14] Sai Q., Bi J., Xie D., & Guan W. Identifying and predicting the expenditure level characteristics of car-sharing users based on the empirical data. Sustainability. 2019; 11:6689.

[15] Shaheen S., & Rodier C. Travel effects of a suburban commuter carsharing service: CarLink case study. Transportation Research Record: Journal of the Transportation Research Board. 2005; 1927:182–188.

[16] Shaheen S.A., & Lipman T.E. Reducing greenhouse emissions and fuel consumption: Sustainable approaches for surface transportation. IATSS Res. 2007; 31:6–20.

[17] Nykvist B., & Whitmarsh L. A. Multi-level analysis of sustainable mobility transitions: Niche development in the UK and Sweden. Technol. Forecast. Soc. Chang. 2008; 75:1373–1387.

[18] Rodier C. Review of international modeling literature: Transit, land use, and auto pricing strategies to reduce vehicle miles traveled and greenhouse gas emissions. Transportation Research Record: Journal of the Transportation Research Board. 2009; 2132:1–12.

[19] Park G., Lee S., Jin S., & Kwak S. Integrated modeling and analysis of dynamics for electric vehicle powertrains. Expert. Syst. Appl. 2014; 41:2595–2607.

[20] Sierzchula W. Factors influencing fleet manager adoption of electric vehicles. Transp. Res. Part D: Transp. Environ. 2014; 31:126–134.

[21] Navigant Research. Carsharing services will surpass 12 million members worldwide by 2020. Accessed August 22, 2013. https://www.navigantresearch.com/newsroom/carsharing-services-will-surpass-12-million-members-worldwide-by-2020.

[22] Zhou Y., Li Y., Hao M., & Yamamoto T. A System of shared autonomous vehicles combined with park-and-ride in residential areas. Sustainability. 2019; 11:3113.

[23] De Luca S., & Di Pace R. Modelling users’ behaviour in inter-urban carsharing program: A stated preference approach. Transport. Res. Part A. 2015; 71:59–76.

[24] Rouan R. Car2go shrinks its service area, The Columbus Dispatch, 2015-8-10. http://www.dispatch.com/content/stories/local/2015/08/10/Car2Go_shrinks_area.html

[25] Duan Q., Ye X., Li J., & Wang K. Empirical modeling analysis of potential commute demand for carsharing in Shanghai, China. Sustainability. 2020; 12(2):620.

[26] Porter M. E. Competitive strategy; techniques for analyzing industries and competitors. 1980; New York, Free Press.

[27] Lamberton C. P., & Rose R. L. When is ours better than mine? A framework for understanding and altering participation in commercial sharing systems. Journal of Marketing. 2012; 76:109–125.

[28] Burlando C., Ivaldi E., Saiani P., & Penco L. To own or not to own? Car ownership and consumer awareness: Evidence from an Italian survey. Research in Transportation Business & Management. 2020; January:100435.

[29] Millard-ball A., Murray G., & Schure J. T., et al. Car sharing: Where and how it succeeds. In Tcrp Report Transportation Research Board of the National Academies. 2005, USA.

[30] Burkhardt J., & Millard-ball A. Who is attracted to carsharing? Transportation Research Record: Journal of the Transportation Research Board. 2006; 1986:98–105.

[31] Kim D., Ko J., & Park Y. Factors affecting electric vehicle sharing program participants’ attitudes about car ownership and program participation. Transportation Research Part D: Transport and Environment. 2015; 36:96–106.

[32] Hui Y., Sun Q., & Sun T. Study on the influencing factors of new-type car ownership choice. China Transportation Review. 2017; 8:61–65+114.(in Chinese)

[33] Hui Y., Sun Q., Ding M., & Wang W. An analysis on trip characteristics and utility of carsharing members: A case study of Chefenxiang in Hangzhou. Shanghai Urban Planning Review. 2018; 139:26–32. (in Chinese)

[34] Litman T. Evaluating carsharing benefits, Transportation Research Record: Journal of the Transportation Research Board. 2000; 1702:31–35.

[35] Ciari F, Weis C, & Balac M. Evaluating the influence of carsharing stations’ location on potential membership: a Swiss case study. Euro Journal on Transportation & Logistics. 2016; 5(3):345–369.

[36] Wolf J., Guensler R., & Bachman W. Elimination of the travel diary: experiment to derive trip purpose from global positioning system travel data. Transportation Research Record Journal of the Transportation Research Board. 2001; 1768:125–134.

[37] Wang Y., Correia G., Arem B., & Timmermans H. J. P. Understanding travellers’ preferences for different types of trip destination based on mobile internet usage data, Transportation Research Part C. 2018; 90:247–259.

[38] Liu J., Xu J., & Cai L., et al. Identifying functional regions based on the spatio-temporal pattern of taxi trajectories. Journal of Geo-information Science. 2018; 20:1550–1561. (in Chinese)

[39] Wang, J., Gao, F., Cui, P., Li, C., & Xiong, Z. Discovering urban spatio-temporal structure from time-evolving traffic networks. In: Asia-pacific Web Conference. 2014, Springer International Publishing.

[40] Alexander L., Jiang S., Murga M., & González M.C. Origin-destination trips by purpose and time of day inferred from mobile phone data. Transp. Res. Part C. 2015; 58:240–250.

[41] Wang Y., Ma X. L., Liu Y., Gong K., Xu M. Z., Wang Y. H. A Two-Stage Algorithm for Origin-Destination Matrices Estimation Considering Dynamic Dispersion Parameter for Route Choice. Plos One, 2016; 11(1): e0146850. doi: 10.1371/journal.pone.0146850 26761209 PMC4712001

[42] Wang Y., Ma X. L., Li Z. B., Liu Y., Xu M. Z., Wang Y. H. Profit Distribution in Collaborative Multiple Centers Vehicle Routing Problem. Journal of Cleaner Production, 2017; 144: 203–219.

[43] Wang Y., Peng S. G., Zhou X. S., Mahmoudi M., Zhen L. Green logistics location-routing problem with eco-packages. Transportation Research Part E: Logistics and Transportation Review, 2020; 143:102118.

[44] Wang Y., Assogba K., Fan J. X., Xu M. Z., Liu Y., Wang H. Z. Multi-depot green vehicle routing problem with shared transportation resource: Integration of time-dependent speed and piecewise penalty cost. Journal of Cleaner Production, 2019; 232: 12–29.

[45] Jiang S., Alves A., Rodrigues F., Ferreira J.C. Jr., & Pereira F. Mining point-of-interest data from social networks for urban land use classification and disaggregation. Comput. Environ. Urban Syst. 2015; 53:36–46.

[46] Phithakkitnukoon S., Horanont T., Lorenzo G. Di, Shibasaki R., & Ratti C. Activity-aware map: identifying human daily activity pattern using mobile phone data. In: Human Behavior Understanding. 2010, Springer, 14–25.

[47] Yuan, J., Zheng, Y., & Xie, X. Discovering regions of different functions in a city using human mobility and POIs. In: Proceedings of the 18th ACM SIGKDD International Conference on Knowledge Discovery and Data Mining—KDD ‘12. ACM Press, 2012, New York, USA.

[48] Demissie M.G., Correia G., Bento C. Analysis of the pattern and intensity of urban activities through aggregate cellphone usage. Transp. A Transp. Sci. 2015; 11:502–524.

[49] Resnick, P., Iacovou, N., Suchak, M., Bergstrom, P., & Riedl, J. GroupLens: an open architecture for collaborative filtering of netnews. In: Proceedings of the 1994 ACM Conference on Computer Supported Cooperative Work—CSCW ‘94. ACM Press, 1994, New York, USA.

[50] Xue, G.-R., Lin, C., Yang, Q., Xi, W., Zeng, H.-J., Yu, Y., et al. Scalable collaborative filtering using cluster-based smoothing. In: Proceedings of the 28th Annual International ACM SIGIR Conference on Research and Development in Information Retrieval—SIGIR ‘05. ACM Press, 2005, New York, USA.

[51] Everitt B.S., Landau S., Leese M., & Stahl D. Hierarchical Clustering. John Wiley & Sons, Ltd, 2011, 71–110.

[52] Sørensen T. A method of establishing groups of equal amplitude in plant sociology based on similarity of species and its application to analyses of the vegetation on Danish commons. Biol. Skr. 1948; 5:1–34.

[53] Dunn J. C. A fuzzy relative of the ISODATA process and its use in detecting compact well-separated clusters. Journal of Cybernetics. 1973; 3:32–57.

[54] Lu Q.C. Modeling network resilience of rail transit under operational incidents, Transp. Res. A. 2018; 117:227–237.

[55] Zhao D., Wang W., Ong G.P., & Ji Y. An association rule based method to integrate metro-public bicycle smart card data for trip chain analysis, J. Adv. Transp. 2018;.

[56] Guo X., Wang D.Z.W., & Wu J., et al. Mining commuting behavior of urban rail transit network by using association rules. Physica A: Statal Mechanics and its Applications. 2020; 559:0378–4371.

